# The nonsense mutation of the *smu-1(mn415)* allele is in exon 6 at Q257, not in exon 5 at Q255

**DOI:** 10.17912/micropub.biology.002454

**Published:** 2026-10-22

**Authors:** Nirmala Devi Rajendra Chauhan, Timothy Pearson, Alper Akay

**Affiliations:** 1 School of Biological Sciences, University of East Anglia, Norwich, ENG, United Kingdom

## Abstract

*smu-1* encodes the *Caenorhabditis elegans* orthologue of the spliceosomal protein SMU1. The *smu-1 ( mn415 )* allele was originally identified as a suppressor of synthetic lethality between *mec-8* and *unc-52* and was mapped to a stop codon at amino acid position 255 in exon 5. Our Sanger sequencing analysis of the *smu-1 ( mn415 )* allele shows that the stop codon is at amino acid position 257 in exon 6.

**Figure 1. smu-1(mn415) has a Gln > STOP mutation in exon 6:** (A) Diagram of *smu-1* exons 5 and 6. Exon sequences and the corresponding amino acid sequences are shown in letters. The previously reported and the current position of the stop codon are shown with an arrow. (B) Gel electrophoresis of PCR products around Gln255 in wild-type (WT) and *smu-1 ( mn415 )* containing SP1792 strain with and without restriction digestion with SspI (-/+ RE). (C) Sanger sequencing alignment traces of PCR products around Gln255. (D) Sanger sequencing alignment traces of PCR products around Gln257. (E) Gel electrophoresis of PCR products around Gln257 in wild-type (WT) and SP1792 strain with and without restriction enzyme digestion with DdeI (-/+RE; the wild-type amplicon has an additional DdeI site observed as a small shift in the WT +RE band). Yellow arrows indicate unspecific / primer dimer bands.

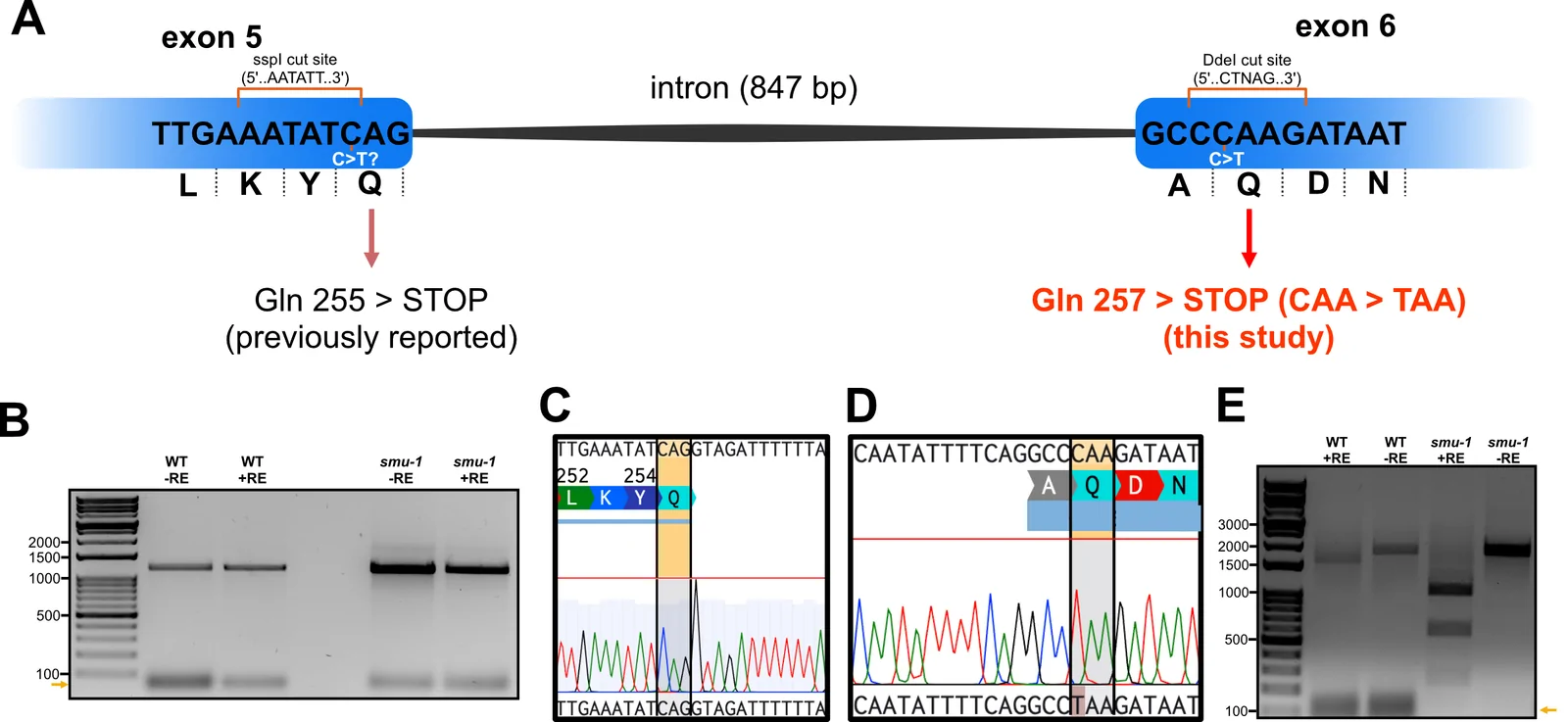



## Description

SMU1/fSAP57 and SMU2/RED are components of the spliceosomal B complex and interact structurally (Hegele et al., 2012; Keiper et al., 2019; Spartz et al., 2004; Zhang et al., 2024). Both proteins are required during B complex activation and for efficient splicing of predominantly short introns (Keiper et al., 2019). Mutations in either gene cause splicing defects in multiple organisms (Chung et al., 2009; Dassah et al., 2009; Keiper et al., 2019; Spartz et al., 2004; Spike et al., 2001; Sugaya et al., 2006). The *smu-1 ( mn415 )* allele was originally identified as a suppressor of the synthetic lethal *mec-8 (u218ts); unc-52 ( e669su250 ts)* double mutants (Lundquist & Herman, 1994). Later studies showed that *smu-1 ( mn415 )* and other *smu-1* and *smu-2* alleles cause alternative splicing of genes harbouring intronic mutations, which contributes to genetic suppression of mutant phenotypes (Dassah et al., 2009; Spartz et al., 2004; Spike et al., 2001).

Molecular characterisation of the *smu-1 ( mn415 )* allele reported a CAA to TAA transition that converts glutamine 255 in exon 5 into a stop codon ( Figure 1A ) (Spike et al., 2001). We noted that the Gln255 codon is CAG, not CAA, and therefore assumed the reported stop codon could be TAG rather than TAA. While genotyping the strain SP1792 *smu-1 ( mn415 )* I; *unc-52 ( e669su250 )* II, our attempts to use the restriction enzyme SspI (cuts at 5ʹ…AAT/ATT…3ʹ), predicted to digest the PCR product around Gln255, failed. Sanger sequencing of the PCR product around Gln255 also did not reveal any mutations on or around Gln255 ( Figure 1B ). Gln255 is the last amino acid of exon 5, which is separated from exon 6 by an 847 bp intron ( Figure 1A ). We also noted that the second amino acid of exon 6 (amino acid 257) is Gln, with the CAA codon. Therefore, we PCR-amplified and Sanger-sequenced the region around Gln257, which revealed a C to T transition converting Gln257 into a TAA stop codon ( Figure 1C ). We confirmed that the CAA to TAA mutation at Gln257 is digested by the restriction enzyme DdeI (cuts at 5ʹ…C/TNA/G…3ʹ) ( Figure 1D ).

We do not expect the new position of the stop codon in *smu-1 ( mn415 )* to alter any reported phenotypes. Nevertheless, these results could help other researchers genotype *smu-1 ( mn415 )* more easily *.*

## Methods

Strains used in this study:

**Table:** 

**Strain name**	**Genotype**	**Source**
N2 Bristol	Wild-type	CGC
SP1792	*smu-1 ( mn415 )* I; *unc-52 ( e669su250 )* II	CGC

*C. elegans* were maintained on NGM plates at 20℃and fed *E. coli* HB101 . *C. elegans* were lysed using the Extract-N-Amp Tissue kit (Sigma, XNAT2). Worms were picked into 10 µl of extraction solution mixed with 2.5 µl of tissue suspension solution. The mixture was heated to 55℃ for 20 min, then to 95℃ for 3 min, and finally mixed with 10 µl of neutralisation solution. For amplification of the Gln255 amplicon, we used the primer set A882 5ʹ-TTTGTAGCCAGTCAACCACTTTC-3ʹ and A883 5ʹ-AGGTACtgtaggtatacggtaag-3ʹ, and for the Gln257 amplicon we used the primer pairs A882 and A904 5ʹ-ACTCGAGTGCCCACGCATTTCC-3ʹ. PCR reactions were carried out using PCRBio TaqMix with an annealing temperature of 62℃. Restriction enzyme digestion with SspI (NEB, cuts at 5ʹ…AAT/ATT…3ʹ) and DdeI (NEB) was set up in a 10 µl volume with NEB Cutsmart buffer. PCR products and restriction digests were run on a 1.5% agarose gel in 1X TAE buffer.
